# COVID-19: Failure of the DisCoVeRy Clinical Trial, and Now–New Hopes?

**DOI:** 10.3390/ph14070664

**Published:** 2021-07-11

**Authors:** Jean Jacques Vanden Eynde

**Affiliations:** Formerly Head of the Department of Organic Chemistry (FS), University of Mons-UMONS, 7000 Mons, Belgium; jean-jacques.vandeneynde@ex.umons.ac.be

**Keywords:** antibodies, COVID-19, cytokine storm, hydroxychloroquine, lopinavir, remdisivir, ritonavir, SARS-CoV-2, stem cells, vaccine

## Abstract

The DisCoVeRy clinical trial aimed at the evaluation of four treatments for patients suffering from severe to critical COVID-19: Hydroxychloroquine, eventually associated with azithromycin; the combination lopinavir/ritonavir; the combination with the addition of interferon β-1a; remdesivir. The trial was discontinued due to the lack of positive results. Meanwhile, many other potential options have been considered either to target the virus itself, the interactions with the host cells, or the cytokine storm frequently observed during the infection. Several of those options are briefly reviewed. They include vaccines, small molecules, antibodies, and stem cells.

## 1. Introduction

Considered as one of the worst pandemics after the Spanish flu, COVID-19 is a respiratory disease caused by a coronavirus (SARS-CoV-2), which could have emerged in humans in November–December 2019 [1,2]. First detected in Wuhan (China), SARS-CoV-2 surprised physicians and healthcare workers by the rapidity and worldwide extent of its dissemination.

Briefly, the interactions between SARS-CoV-2 and host cells are governed by the receptor-binding domain of an external glycoprotein, spike protein or S protein, of the virus and a human cellular receptor, the angiotensin-converting enzyme 2 or ACE2. S protein cleavage is then required to allow viral entry into host cells. That proteolytic cleavage is performed by the serine protease TMPRSS2 and, to a lesser extent, by the cysteine proteases cathepsin B and L (CatB/L). In the cascade of events involved in the replication of the virus, let us point out the essential role played by several viral proteins that actually constitute relevant drug targets: two polyproteins, pp1a and pp1ab, and the RNA-dependent RNA polymerase (RdRp), which are replicase proteins; the chymotrypsin-like protease 3 (3CL^pro^ or main protease M^pro^) and the papain-like protease (PL^pro^), which are cysteine proteases [3,4]. Importantly, human analogs of the coronavirus 3CL^pro^ have not been reported, enabling an exceptional selectivity of its inhibitors [5].

In order to accelerate the availability of medical treatments, the logical approach was to repurpose existing drugs in order to avoid long procedures of phase studies. In that sense, the SOLIDARITY Response Fund was created, in March 2020, by the World Health Organization (WHO), the United Nations Foundation, and the Swiss Philanthropy Foundation [6]. The fund was and is still used to support adaptive international clinical trials aimed at the evaluation of “additional treatments for COVID-19 in hospitalized patients who are all receiving the local standard of care” [7,8,9,10,11]. In the beginning, four options were considered:

- Hydroxychloroquine, eventually associated with the antibiotic azithromycin;
- The combination lopinavir/ritonavir;
- The combination lopinavir/ritonavir with the addition of interferon β-1a;
- Remdesivir.

## 2. The DisCoVeRy Clinical Trial

DisCoVeRy [12,13] is a European study supported by the SOLIDARITY fund. It was launched by the French Institut National de la Santé Et de la Recherche Médicale (INSERM) in March 2020. In two previous publications, we gave an overview of that trial [14] and the first update of its evolution around two months after its starting date [15].

More than one year has passed now, a good time, in our opinion, to take a look at the results and perspectives.

The DisCoVeRy trial had a study start date on 22 March 2020, and, originally, it was supposed [12] to involve an estimated enrolment of 3200 patients distributed in eight countries: Belgium, France, Germany, Luxemburg, the Netherlands, Spain, Sweden, and the United Kingdom. In fact [13], most investigations were performed in France, in 40 hospitals. Five countries did not take part in that study: Germany, the Netherlands, Spain, Sweden, and the United Kingdom. Belgium and Luxemburg were poorly represented with three and two hospitals, respectively. Austria and Portugal joined the project with the participation of three and four hospitals, respectively [13]. As of 22 July 2020, 801 patients had been included in the trial, following the study chair [16].

### 2.1. Hydroxychloroquine, Eventually Associated with Azithromycin

**Figure 1:**
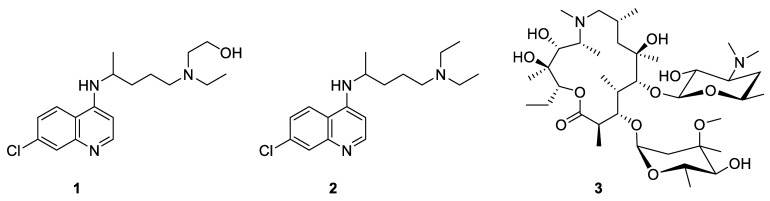
Chemical structure of hydroxychloroquine (**1**), chloroquine (**2**), and azithromycin (**3**).

The first evidence for the efficacy of hydroxychloroquine (**1**, Figure 1), and its structurally related analog chloroquine (**2**, Figure 1), in the treatment of COVID-19 were already reported by Gao et al. [17], in February 2020. A synergetic effect was expected when using hydroxychloroquine in combination with azithromycin (**3**, Figure 1) for preventing bacterial superinfection [18]. However, the usefulness of those drugs has been the subject of many contradictory results. Let us mention, among many others, a highly mediatized debate in France [18,19].

The modes of action of hydroxychloroquine in the treatment of COVID-19 appear to be complex and diverse, going from inhibition of the binding between the S protein and ACE2 to repression of activation of cytokines further to an increase in the pH of endosomes and lysosomes [20,21,22].

Following the U.S. National Library of Medicine of the National Institutes of Health [13,23], the study in the DisCoVeRy trial was stopped on 24 May 2020. Amazingly, a Belgian team [24] analyzed a set of data concerning 15,544 patients hospitalized in their country over a period ending on the same day! Moreover, the report concluded that a significant decrease in the number of deaths was observed in the group (4542 patients) having been treated with hydroxychloroquine.

### 2.2. The Combination Lopinavir/Ritonavir and the Combination with the Addition of Interferon β-1a

**Figure 2:**
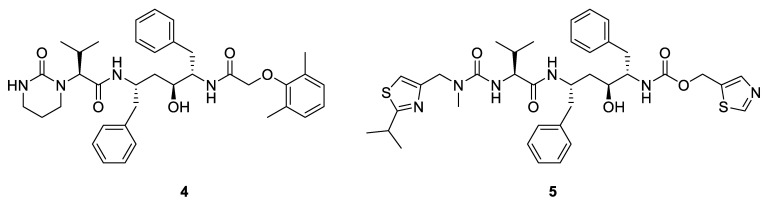
Chemical structure of lopinavir (**4**) and ritonavir (**5**).

The combination of lopinavir (**4**, Figure 2) and ritonavir (**5**, Figure 2) is commercially available under the brand name Kaletra^®^. It was approved by the Food and Drug Administration (FDA) in September 2000 for the treatment of HIV-1 infection in adults and children older than 6 months [25]. In the case of COVID-19, the combination was evaluated as soon as January 2020 in a hospital in Wuhan (China). The trial involved 99 patients who did not need any respiratory support. The investigators did not find any advantage in using the combination when compared with a group of 100 patients who received standard of care alone [26]. Since then, it was demonstrated that the combination lopinavir/ritonavir targeted the 3C–like protease of SARS-CoV-2 [27].

Interferon β-1a is known to be an antiviral agent with a broad spectrum of action and there are many data [28,29,30] on its in vitro activity against coronaviruses responsible for severe acute respiratory syndrome (SARS) and the Middle East respiratory syndrome (MERS). Interferon β-1a is also an essential modulator of the immune system; it is one of the first proteins released in cells in response to viral and bacterial attacks [31]. However, it was observed that SARS-CoV-2 has the capability of perturbating several pathways yielding that interferon [32,33,34], thus justifying an external supply.

Interferon β-1a, associated with lopinavir and ritonavir, exhibited some promising results in the treatment of marmosets infected by MERS-CoV [35] as well as in a small group of patients (43) hospitalized with MERS [36]. A decrease in the risk of mortality for patients with confirmed severe COVID-19 was also observed in a recent study [37].

Following the U.S. National Library of Medicine of the National Institutes of Health [13,23], both arms involving the combination lopinavir/ritonavir in the DisCoVeRy trial were stopped on 29 June 2020.

### 2.3. Remdesivir

**Figure 3:**
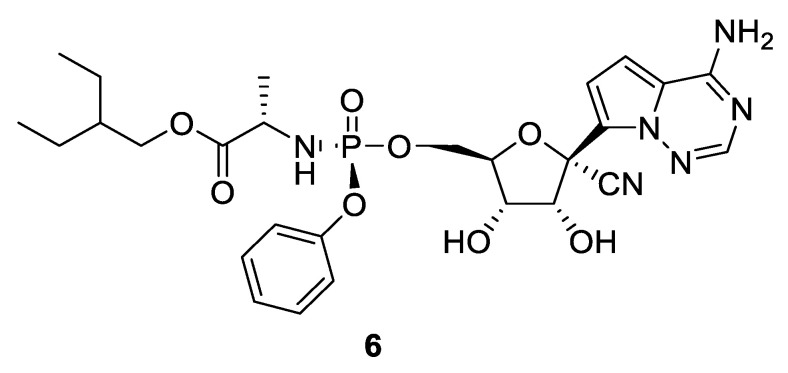
Chemical structure of remdesivir (**6**).

Before the outbreak of SARS-CoV-2, remdesivir (**6**, Figure 3) was poorly studied in clinical trials. To the best of our knowledge, it had been the subject of one study only [23,38]. It was used for the treatment of people with Ebola virus disease in 2018 [38]. Mention must be made that the results were highly disappointing. Indeed, the highest number of deaths was observed in the group treated with the drug and exceeded 50% of the patients in the arm [38,39]. Consequently, it was impossible to measure any decrease in the viral load potentially induced by remdesivir.

It should be remembered that, in January 2020, the first patient suffering from COVID-19 in the United States was successfully treated with remdesivir in a hospital in the State of Washington [40]. That result was followed by the implementation of an international clinical trial in 60 locations around the world, among which 41 were in the USA. The trial, named Adaptive COVID-19 Treatment Trial (ACTT) [41], led to the conclusion, “that remdesivir (517 patients) was superior to placebo (508 patients) in shortening the time to recovery in adults who were hospitalized with COVID-19 and had evidence of lower respiratory tract infection” [42]. Further to the publication of the preliminary report on 8 October 2020, the drug was approved, on 22 October 2020, by the FDA for the treatment of COVID-19 requiring hospitalization [43]. However, the approval package mentioned that clinical trials were still required by the FDA in order to administer the drug to people younger than 18, to determine side effects, and to study its efficacy against variants of SARS-CoV-2.

On the other hand, on 27 January 2021, the INSERM announced [44] that patients’ recruitment for the arm involving remdesivir in the DisCoVeRy trial had been suspended since 19 January 2021 [13]. The decision was based on an interim report describing observations obtained on a group of 389 patients that had received the drug. Nevertheless, in July 2021, the European Medicines Agency (EMA) followed the attitude of the FDA and authorized the use of remdesivir to treat COVID-19 [45].

Regarding the mode of action in the case of SARS-CoV-2, it was established that remdesivir is an inhibitor of the RNA-dependent RNA polymerase and, potentially, also of the 3C–like protease [46,47,48].

### 2.4. The Conclusions of the DisCoVeRy Trial

Data collected during the trial have not yet been published [18]. However, they were included in the interim WHO Solidarity trial results published on 11 February 2021 [49]. The report concluded: “These remdesivir, hydroxychloroquine, lopinavir, and interferon regimens had little or no effect on hospitalized patients with COVID-19, as indicated by overall mortality, initiation of ventilation, and duration of hospital stay”.

In the case of remdesivir, results of the ACCT trial and FDA approval led the WHO to moderate its position by suggesting, “a conditional recommendation against remdesivir in hospitalized patients with COVID-19”. In the cases of hydroxychloroquine and the combination lopinavir/ritonavir, the WHO emitted “a strong recommendation against those drugs in patients with COVID-19 of any severity” [50].

## 3. New Hopes?

Besides the treatments initially considered by the SOLIDARITY fund, many other opportunities have emerged as potential hopes to fight COVID-19. Some of them, the most highlighted to the public, are briefly described hereafter. Evolution of the number of clinical trials registered in the U.S. National Library of Medicine of the National Institutes of Health [23] over the past 14 months for a given potential therapy, as reported in Table 1, gives a good idea of the interest it creates.

As far as possible, within each section below, drugs are listed in alphabetical order.

### 3.1. Vaccines

In order to prepare the immune system to efficiently respond to a SARS-CoV-2 infection, vaccination emerged as the most promising and the most privileged tool [51,52]. As indicated in Table 1, the number of studies has been continuously increasing for more than 1 year. Three strategies have been considered:

- Use of mRNA encoding a fragment of the spike protein;
- Use of a harmless virus that will produce spike proteins and consequently induce the immune response;
- Use of spike proteins themselves.

Among the six vaccines actively considered in Western countries (Table 2), the Sanofi/GSK product (still in clinical trials) is the sole vaccine requiring an adjuvant, the GSK AS03 adjuvant manufactured by GSK. The adjuvant contains squalene, DL-α-tocopherol, and polysorbate [53].

Actually, experts agree on the fact that vaccination does not prevent transmission of the virus but limits the manifestations of the disease. However, many points are still awaiting some clarifications, among which duration of the immunization [54]; efficacy against variants of concern [55]; side effects at short, mid, and long term [56]; transmission rate after administration [57]; age of the population to vaccinate [58].

Nevertheless, following the Reuters agency, on 15 June 2021, at least 199 countries have started vaccinating against COVID-19 and more than 2.4 billion doses have been injected [59]. Regarding the number of doses, let us mention that, as of 20 May 2021, the European Union had secured the purchase of 4.4 billion shots. They are distributed among manufacturers as indicated in Table 2 [60]. The price paid per dose by the European Union was unveiled by an indiscretion from a Belgian politician [61].

**Table 2:** Vaccines ordered by the European Union (EU): quantity and price [60,61].

Vaccine (Manufacturer)	Type of Vaccine	Number of Shots	Number of Doses Secured by the EU	Price per Dose in Euros (Tax Excluded)
Vaxzevria (AstraZeneca/Oxford)	Viral vector	2	400 million	1.78
Comirnati (BioNTech/Pfizer)	mRNA	2	2.4 billion	12.00
CVnCoV (CureVac)	mRNA	2	405 million	10.00
Johnson & Johnson (Janssen)	Viral vector	1	400 million	7.08
Spikevax (Moderna)	mRNA	2	460 million	15.00
VAT00008 (Sanofi/GSK)	Protein-vector	2	300 million	7.56

### 3.2. Small Molecules

It is worth mentioning that, in the frame of the DisCoVeRy trial, all evaluated small molecules were antiviral agents and they were all administered to hospitalized patients, most of them having been categorized as severely ill. At that stage of the illness, decreasing the viral load is not sufficient because, in most cases, it is an excessive immune response (cytokine storm) that is responsible for the degradation of health and eventually a fatal outcome. Therefore, three therapeutic strategies are globally possible:

- Early inhibition of the interaction virus-host cells;
- Action on the virus replication;
- Late control of the cytokine storm.

#### 3.2.1. Inhibition of the Interaction Virus-Host Cells

**Figure 4:**
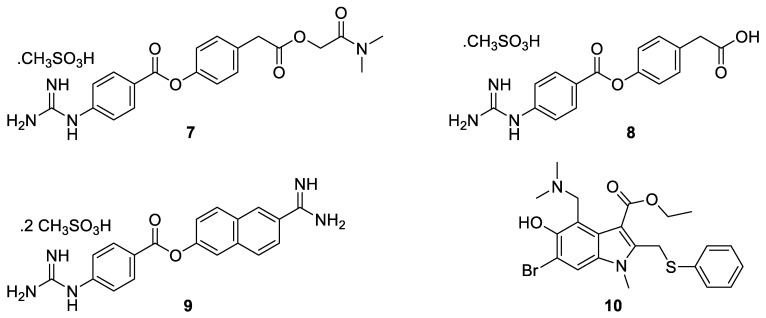
Chemical structure of camostat mesylate (**7**), its metabolite (**8**), nafamostat (**9**), and umifenovir (**10**).

Camostat mesylate (**7**, Figure 4) is a serine protease inhibitor marketed in Japan since 2006 for the treatment of chronic pancreatitis [62,63]. Kawase et al. [64] as well as Zhou et al. [65], among others, had already observed that the drug was able to inhibit the growth of several coronaviruses, including SARS-CoV, by interacting with the spike protein. The idea was further exploited by Hoffmann et al. [3] who demonstrated that the entry of SARS-CoV-2 can be blocked by camostat mesylate in vitro and in vivo. The blockade was attributed to an interaction with the human protease TMPRSS2 producing a decrease in the activity of the enzyme. That led to the ironic title of a post in ScienceMag: “These drugs don’t target the coronavirus—they target us” [66].

In a more recent publication [67], it was reported that, in vivo, camostat mesylate is rapidly hydrolyzed to form 4-(4-guanidinobenzoyloxy) phenylacetic acid (**8**, Figure 4), a metabolite that is also active against SARS-CoV-2.

Hoffmann et al. [68] and Ko et al. [69] evaluated the inhibitory effect of nafamostat (**9**, Figure 4), a structural analog of camostat, which is a non-FDA approved serine protease inhibitor also. The drug is more efficient than camostat, in vitro, with EC_50_ values in the low nanomolar range.

Actually, there are few results on the efficacy of camostat and nafamostat for the treatment of COVID-19. Conclusions from large trials are impatiently awaited.

Umifenovir (**10**, Figure 4) is known under the brand name Arbidol^®^ (hydrochloride salt). It is commercially available, at low cost, in Russia and China for the treatment of influenza. Umifenovir is an indole derivative exhibiting good efficacy against a large panel of viruses [70]. In the case of SARS-CoV-2 (multiplicity of infection of 0.05), Wang et al. determined an EC_50_ of 4.11 μM when **10** was tested in a culture of infected African green monkey kidney cells, Vero E6 (ATCC-1586) [71]. Following molecular dynamics simulations, umifenovir targets the virus by exhibiting a higher affinity than the angiotensin-converting enzyme 2 for the receptor-binding domain of the spike protein [72,73]. Because the drug is not approved by the FDA and is of limited access worldwide, the substance is poorly involved in large clinical trials and some contradictory results, obtained on small cohorts, have been reported [74,75,76].

#### 3.2.2. Action on the Virus Replication

**Figure 5:**
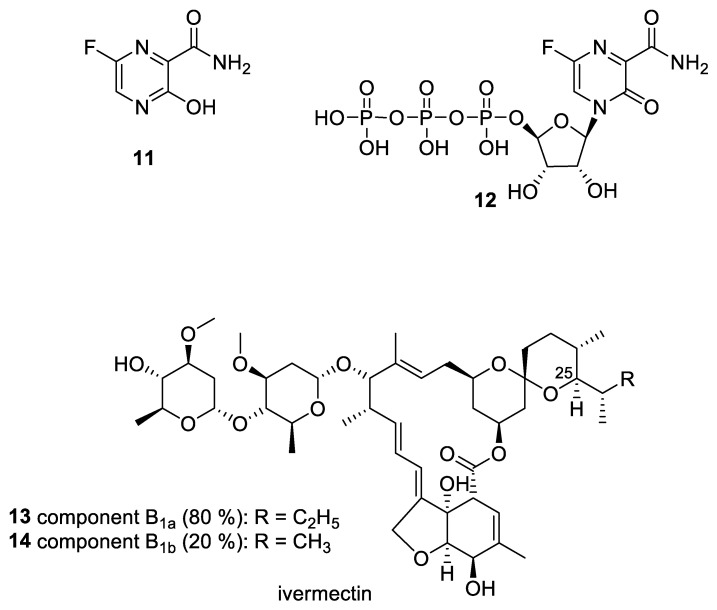
Chemical structure of favipiravir (**11**), its metabolite **12**, and ivermectin (**13**–**14**).

Favipiravir (**11**, Figure 5) is a pyrazinecarboxamide commercialized in Japan, under the name Avigan^®^, for the treatment of influenza. It acts as a prodrug: after a metabolic transformation into its 4-ribonucleoside 5′-triphosphate form (**12**, Figure 5), it inhibits the RNA-dependent RNA polymerase (RdRp) [77,78]. The drug exhibited an anti-SARS-CoV-2 efficacy in hamster models but at high doses [79,80]. Despite the poor availability of the drug in Western countries, favipiravir is seeing a renewed interest in recent months (see Table 1). In that way, its efficacy at an early stage of the disease is evaluated in France under the title “Home treatment of elderly patients with symptomatic SARS-CoV-2 infection (COVID-19: a multiarm, multi-stage (MAMS) randomized trial to assess the efficacy and safety of several experimental treatments to reduce the risk of hospitalization or death” [81]. A similar trial was launched in the USA and Canada, with the approval of the FDA. It aims at determining the potential of favipiravir as a chemoprophylactic agent or a treatment agent in long-term care homes [82]. The wave was followed in April 2021 by the National Institute for Health Research (UK), which added favipiravir to the panel of drugs involved in the PRINCIPLE clinical trial [83]. The study focuses on a possible treatment for patients recovering at home [84].

Ivermectin is made of two components differing by one methylene group in the side chain in position 25 of the skeleton (**13** and **14**; Figure 5). The substance was approved by the FDA in 1996 as an anthelmintic agent [85]. More recent works reported an effective in vitro capability of ivermectin to reduce the replication of several viruses, including SARS-CoV-2 [86,87,88,89,90].

Despite encouraging results for prophylaxis and treatment in clinical trials [91,92,93], the WHO recommends “not to use ivermectin in patients with COVID-19 except in the context of a clinical trial.” [50] The recommendation was justified by “the high degree of uncertainty in the most critical outcomes such as mortality and need for mechanical ventilation”. The substance remains, however, of interest, as revealed by the numbers indicated in Table 1.

#### 3.2.3. Control of the Cytokine Storm

**Figure 6:**
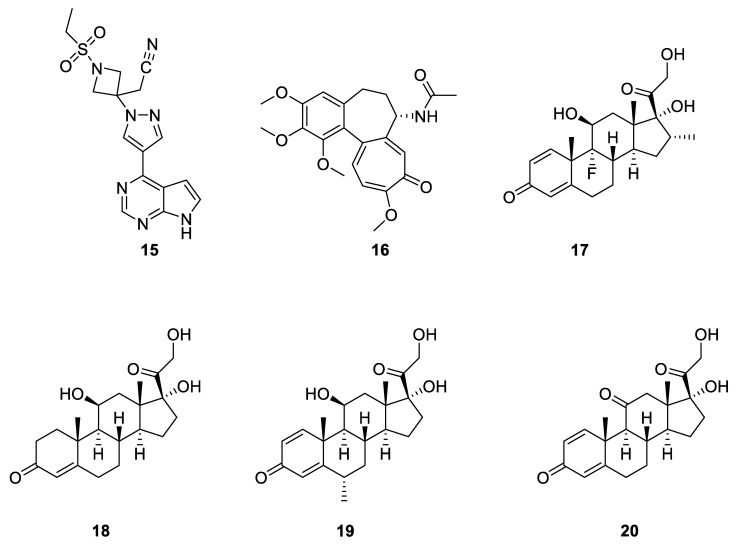
Chemical structure of baricitinib (**15**), colchicine (**16**), dexamethasone (**17**), hydrocortisone (**18**), methylprednisone (**19**), and prednisone (**20**).

Baricitinib (**15**, Figure 6) is the active ingredient of Olumiant^®^, commercialized by Eli Lilly and Co (Indianapolis, IN, USA). It was approved by the FDA on 31 May 2018 for the treatment of rheumatoid arthritis [94,95,96]. The drug is a selective and reversible inhibitor of JAK1 and JAK2, which acts by blocking some cytokine pathways. The COV-BARRIER clinical trial [97] is a clinical trial sponsored by Incyte Co (Alapocas, DE, USA) and Eli Lilly and Co, the developers of baricitinib. In April 2021, they disclosed results indicating that baricitinib did not improve the development of COVID-19 for hospitalized patients with severe illness when compared to a group receiving a placebo and standard of care. However, the study indicated that a significant reduction of death (38%) was observed in the arm of patients receiving baricitinib in addition to corticosteroids and/or remdesivir [98,99]. Those combinations are now recommended by the US National Institutes of Health [100]. The efficacy of the combination baricitinib/remdesivir had been already observed in another clinical trial (ACTT-2) funded by the National Institute of Allergy and Infectious Diseases (USA) [101,102]. Encouraged by those results, the EMA is evaluating the possibility of including baricitinib in the potential treatments of COVID-19 in European hospitals [103].

Colchicine (**16**, Figure 6) is a plant alkaloid used for centuries because of its anti-inflammatory properties. It is widely prescribed in the treatment of gouty arthritis. In April 2020, it was suggested to consider colchicine as a potential drug in cases of COVID-19 [104]. Besides encouraging results demonstrating that, “colchicine reduced the length of both supplemental oxygen therapy and hospitalization”, the investigators of a small clinical trial in Brazil admitted that, “it is not possible to ensure that colchicine reduced mortality of COVID-19” [105]. On the other hand, the investigators of the Canadian trial COLCORONA [106] reported, in a preprint, that, “among non-hospitalized patients with COVID-19, colchicine reduces the composite rate of death or hospitalization” [107]. The study was performed on a group of 2235 patients receiving colchicine and a group of 2253 patients receiving a placebo. In both groups, the median age was 54 ± 10 years. It is noteworthy that the data focused on non-hospitalized people, contrary to most other trials. On 3 March 2021, the National Institute for Health Research (UK) decided to include colchicine in an arm of its PRINCIPLE clinical trial [83]. The drug might be administered to patients aged 18–64 with moderate symptoms of recent COVID-19 infection and to participants aged over 65 [108]. Despite those hopes, a statement of the chief investigators of the RECOVERY trial, published on 5 March 2021, underlined that “To date there has been no convincing evidence of the effect of colchicine on clinical outcomes in patients admitted to hospital with COVID-19” [109].

Corticosteroids are well-known for their anti-inflammatory properties [110]. Therefore, because SARS-CoV-2 can trigger a cytokine storm of the host, numerous studies have been dedicated to the evaluation of their efficiency in the treatment of COVID-19. Dexamethasone (**17**, Figure 6), hydrocortisone (**18**, Figure 6; the synthetic analog of the naturally produced cortisol), methylprednisolone (**19**, Figure 6), and prednisone (**20**, Figure 6) are among the derivatives that received the greatest interest. Among them, dexamethasone emerged as first-line therapy because of its long-lasting action. However, those corticosteroids are recommended for severely ill patients requiring respiratory support only. In addition, the use of corticosteroids was associated with a lower risk of mortality compared with the use of standard care or placebo [111]. The terms “lower risk” reflect the fact that mortality was reduced from 415/1000 for the groups receiving standard care to 328/1000 for the groups receiving corticosteroids [50]. That moderate optimism was confirmed by results collected in the RECOVERY clinical trial [112,113] sponsored by the University of Oxford (UK). Indeed, the study concluded that, “overall, 482 patients (22.9%) in the dexamethasone group (2104 patients) and 1110 patients (25.7%) in the usual care group (4321 patients) died within 28 days after randomization” [114].

### 3.3. Antibodies

#### 3.3.1. Convalescent Plasma

Convalescent plasma transfusion is an option considered to fight COVID-19. However, due to the diversity of the samples, large statistical studies can hardly be realized. Many factors must be considered, among which availability of samples, quality of the blood of the donors (infection-free), transfused volumes, and titers of antibodies [115]. In addition, several mechanisms of action are possible, starting from neutralization of the virus to regulation of the immune responses [116]. 

A rapid survey of the literature indicates that the efficacy of convalescent plasma in the treatment of COVID-19 is based essentially on the exploitation of the high content of antibodies. Consequently, such a therapy should be of particular interest when restricted to an early administration in moderately ill patients [117,118]. Conflicting results were published when convalescent plasma was transfused to hospitalized patients [119,120]. In particular, as part of the RECOVERY clinical trial [112,113], 11,558 patients distributed in 177 UK hospitals received either standard of care alone or standard of care and convalescent plasma. The study, extended from 28 May 2020 to 15 January 2021 concluded: “in patients hospitalized with COVID-19, high-titer convalescent plasma did not improve survival or other prespecified clinical outcomes” [120]. Interestingly, no particular side effect associated with the therapy is expected, except those linked to the transfusion itself.

#### 3.3.2. Monoclonal Antibodies

Monoclonal antibodies may be considered as therapeutic tools in the treatment of COVID-19 either at the early stage of the disease by targeting the spike protein or for hospitalized patients by modulating the cytokine storm [121,122,123].

##### Inhibition of the Interaction Virus-Host Cells

Bamlanivimab (LY-CoV555, LY3819253) alone and in combination (1/2; *w/w*) with etesevimab (LY-CoV016, JS016, LY3832479) are under evaluation in the clinical trial BLAZE-1 [124]. The trial is sponsored by Eli Lilly in collaboration with AbCellera Biologics (Vancouver, BC, Canada) and Junshi Biosciences (Shanghai, China). Preliminary results indicated that the combination only, administered as an IV infusion, is able to reduce the viral load in patients with mild to moderate COVID-19 [125]. A more detailed analysis of the data collected by the group indicated that, in fact, bamlanivimab is more efficacious than etesevimab against the Wuhan variant of SARS-CoV-2. On the other hand, etesevimab is more efficiacious (than bamlanivimab) against recent variants [125]. Nevertheless, the FDA estimated that the combination is unlikely to be active against the beta (B.1.351; South Africa) and gamma (P.1; Brazil) variants [126]. Consequently, the US government has halted distribution of the monotherapy at the end of March 2021 [127].

Regdanvimab or CT-P59 is manufactured by the Korean company Celltrion Healthcare (Incheon, Korea). The antibody exhibited an anti-SARS-CoV-2 activity, in vitro, with an IC_50_ value of 8.4 ng/mL against a Korean clinical isolate. In vivo studies, performed in ferrets, golden Syrian hamsters, and rhesus monkeys, confirmed the antiviral efficacy [128]. Regdanvimab appeared to be poorly active in vitro against the beta variant but animal experiments clearly indicated a decrease in the viral load in the respiratory tract of infected ferrets [129]. Celltrion Healthcare announced that, further to a Phase 1 clinical trial, regdanvimab is a safe and efficacious treatment for patients with mild COVID-19 [128]. Additional data are still awaited but the company is already working on the possibility of using that antibody in combination with others [130]. At the end of March 2021, the EMA allowed its use, but with caution [131].

REGN-COV2 is certainly the most popular combination of monoclonal antibodies used in the treatment of COVID-19 since it was administered to Donald Trump, former president of the USA, and to some politicians of his party also. REGN-COV2, a combination of casirivimab (REGN10933) and imdevimab (REGN10987), is marketed by Regeneron Pharmaceuticals (Tarrytown, NY, USA). Interim results from a study on 275 patients were published by Weinreich et al. and demonstrated the safety and efficacy of the combination [132]. Because both antibodies target two different regions of the receptor-binding domain of the spike protein, it is expected that the combination will remain efficacious against variants of SARS-CoV-2 [132,133].

Sotrovimab, also known as VIR-7831 and GSK4182136, is a monoclonal antibody developed by Vir Biotechnology, Inc (San Francisco, CA, USA) and GlaxoSmithKline (Brentford, UK). There is little literature on VIR-7831, which is an evolution of an antibody (S309) isolated from a patient who recovered from SARS in 2003 [134]. Interestingly, VIR-7831 appeared to keep its efficacy, in vitro, against the beta (B.1.351; South Africa) and gamma (P.1; Brazil) variants of SARS-CoV-2 [134]. It was granted authorization of emergency use by the FDA [135], further to interim results of the clinical trial COMET-ICE [136]. Progression of COVID-19 was reduced by 85% in a cohort of 291 patients having received the antibody when compared with the cohort (292 patients) having received a placebo. A study, BLAZE-4, aimed at evaluating the potential of a combination with bamlanivimab, is currently in progress [137].

##### Control of the Cytokine Storm

It was established that interleukin 6 (IL-6), a pleiotropic cytokine, is produced at a too elevated level in response to the SARS-CoV-2 infection. Sarilumab and tocilizumab are two monoclonal antibodies that are able to block the receptor of IL-6. Both of them are FDA-approved and prescribed in the treatment of rheumatoid arthritis. Because of their known efficacy, they were rapidly involved in clinical trials for patients hospitalized with severe or critical COVID-19.

Sarilumab (kevzara^®^ by Regeneron Pharmaceuticals and Sanofi (Paris, France)) did not reach the initial expectations. No significant difference was observed between patients receiving the antibody or standard of care. Faster recovery [138] and synergetic effect with the administration of corticosteroids [139] are claimed, but, in general, no significant difference was observed between patients receiving the antibody or standard of care [138,139,140].

As soon as at the end of March 2020, the FDA authorized Genentech (South San Francisco, CA, USA), a member of the Roche group, to conduct a Phase 3 clinical trial for Tocilizumab (actemra^®^, licensed by Hoffmann-La Roche (Basel, Switzerland)) [141]. The study (COVACTA [142]) did not reach all expectations of the company because the antibody could decrease the time of hospitalization but did not improve the status of the patients nor reduce mortality [143]. Many other trials were initiated by Hoffmann-La Roche but, roughly, tocilizumab alone did not constitute an efficient treatment. The antibody was also evaluated in combination with remdesivir (REMDACTA [144]), but without more success [145]. A synergetic effect could be observed when patients receive tocilizumab in addition to a corticosteroid (dexamethasone) [112,113,146]. In their latest COVID-19 treatment guidelines [100], the US National Institutes of Health (NIH) recommended “using tocilizumab in combination with dexamethasone alone or dexamethasone plus remdesivir for the treatment of COVID-19 in hospitalized patients on high-flow oxygen or noninvasive ventilation who have evidence of clinical progression or increased markers of inflammation.” This statement was followed (24 June 2021) by an announcement of the FDA authorizing an emergency use of the antibody for, “the treatment of hospitalized adults and pediatric patients (2 years of age and older) who are receiving systemic corticosteroids and require supplemental oxygen” [147]. Tocilizumab emerges as one of the most investigated monoclonal antibodies, as indicated by the number of ongoing clinical trials involving it (Table 1).

Siltuximab (Sylvant^®^, Janssen Biotech (Horsham, PA, USA)) is a monoclonal antibody that directly blocks interleukin 6. It is poorly studied in the case of COVID-19. Results acquired with an observational cohort of 30 participants in Italy [148,149] opened some hopes but confirmations remain awaited. A combination with corticosteroids is also under study [150].

Anakinra, whose brand name is Kineret^®^, is manufactured by Swedish Orphan Biovitrum (Solna, Sweden) and is known for its anti-inflammatory action in the treatment of rheumatoid arthritis. The antibody, produced in *Escherichia coli*, is a recombinant form of the naturally occurring interleukin-1 receptor antagonist. Its usefulness in the management of the cytokine storm was recently reviewed by Metha et al. [151]. Several studies indicated that anakinra was safe and could reduce the need for ventilation as well as mortality for patients hospitalized with severe COVID-19 [152,153,154,155,156]. On the other hand, its efficacy in the treatment of mild infections is doubtful [156]. However, all authors underlined that more randomized clinical trials are required to confirm those conclusions.

### 3.4. Mesenchymal Stem Cells

Before the outbreak of COVID-19, the therapeutic potential of mesenchymal stem cells in the treatment of lung injuries had been already recognized [157]. The approach has the combined advantages of modulating the immune response (cytokine storm) and regenerating impaired cells [158]. In addition, mesenchymal stem cells are deprived of the angiotensin-converting enzyme 2 (ACE2) and consequently cannot be attacked by SARS-CoV-2 [3]. Several reports in the treatment of severely ill patients are promising, as reported by Mahandiratta et al. [159]. However, such a cell therapy is known to be cost-effective and time-consuming, thus limiting its applicability at large clinical stages.

## 4. Official Status of the Potential Treatments

The most recent official recommendations on potential treatments cited in this manuscript are gathered in Table 3. They were published by the WHO, the FDA, and the EMA.

## 5. Conclusions

Besides curing people at any stage of the disease, the stated goal of governments and health care professionals is to avoid saturation of the intensive care units. Consequently, most efforts were directed to the evaluation of potential treatments for severely ill patients. That led to some failures, similar to that observed in the DisCoVeRy clinical trial. Hydroxychloroquine, for example, and other antiviral agents and antibiotics [178,179] could give satisfactory results in non-severe COVID-19 cases. Otherwise, many substances used in the treatment of rheumatoid arthritis constitute a pool stimulating much interest. Monoclonal antibodies, especially tocilizumab and anakinra (Table 1), also emerged as highly promising therapeutic tools (Table 3), but they are plagued by their price. Nevertheless, today all hopes lie on the herd immunity that could be provided by a massive and worldwide vaccination.

## Figures and Tables

**Table 1:** Evolution of the number of registered clinical trials [23] over the past 14 months for treatments cited in this opinion.

Search Term	As of				
	28 March 2020	28 April 2020	28 September 2020	28 March 2021	28 May 2021
COVID-19	202	997	3479	5170	5619
Hydroxychloroquine	19	148	252	275	276
Azithromycin	16	62	118	126	127
Lopinavir (+ritonavir)	14	44	80	91	93
Interferon	21	42	99	140	153
Remdesivir	9	18	57	93	103
Vaccine	22	71	240	538	673
Camostat	-	2	9	22	24
Nafamostat	-	1	6	7	7
Umifenovir	9	11	14	14	14
Favipiravir	2	12	37	50	51
Ivermectin	2	5	44	65	68
Baricitinib	2	9	14	18	20
Colchicine	2	8	22	31	33
Corticosteroid	12	35	73	100	111
Dexamethasone	4	10	30	62	72
Hydrocortisone	4	6	15	18	20
Methylprednisolone	5	14	40	51	54
Prednisone	2	4	10	13	16
Plasma	42	123	398	559	593
Bamlanivimab	-	-	4	12	13
Etesevimab	-	-	2	4	5
Regdanvimab/CT-P59	-	-	3	3	3
Regn-Cov2	1	1	4	5	6
Casirivimab	-	-	3	7	9
Imdevimab	-	-	3	7	9
Sotrovimab/VIR-7831	-	-	3	5	5
Sarilumab	4	12	16	17	17
Tocilizumab	6	32	69	82	84
Siltuximab	1	3	4	4	4
Anakinra	4	12	24	34	35
Mesenchymal (cells)	11	30	62	77	81

**Table 3:** Official status of some potential treatments for patients with COVID-19.

Treatment	WHO	FDA	EMA
Hydroxychloroquine	a strong recommendation against use in patients with COVID-19 of any severity [50] (31 March 2020)	FDA cautions against use outside of the hospital setting or a clinical trial [160] (1 July 2020)	only to be used in clinical trials or emergency use programs [161] (1 April 2020)
Lopinavir ritonavir	a strong recommendation against use in patients with COVID-19 of any severity [50] (31 March 2020)	No data found	No data found
Remdesivir	a conditional recommendation against use in hospitalized patients [50] (31 March 2020)	approved for adults and pediatric patients requiring hospitalization [43] (22 October 2020)	conditional marketing authorization [45] (3 July 2020)
Ivermectin	not recommended in patients with COVID-19 except in the context of a clinical trial [50] (31 March 2020)	not approved for the prevention or treatment of COVID-19 [162] (26 April 2021)	against use for the prevention or treatment outside randomized clinical trials [163] (22 March 2021)
Baricitinib	in combination; for hospitalized patients on high-flow oxygen or noninvasive ventilation who have evidence of clinical progression or increased markers of inflammation [164] (27 May 2021)	emergency use authorization in combination with remdesivir [165] (19 November 2020)	EMA starts evaluating the use of Olumiant in hospitalized patients requiring supplemental oxygen [103] (29 April 2021)
Dexamethasone	use of systemic corticosteroids rather than no corticosteroids recommended for patients with severe or critical COVID-19 infection, use not recommended for patients with non-severe infection [166] (2 September 2020)	in the list of drugs used for hospitalized patients [167] (13 October 2020)	can be used in patients on oxygen or mechanical ventilation [168] (18 September 2020)
Bamlanivimab etesevimab	use recommended to treat outpatients with mild to moderate COVID-19 who are at high risk of clinical progression [169,170] (24 May 2021)	FDA revokes emergency use authorization [171] (19 April 2021)	can be used in patients not requiring oxygen and at high risk of progressing to severe disease [172] (5 March 2021)
Regdanvimab CT-P59	No data found	application for emergency use authorization submitted [173] (29 March 2021)	can be used in patients not requiring oxygen and at high risk of progressing to severe disease [131] (26 March 2021)
Regn-Cov2	Use recommended to treat outpatients with mild to moderate COVID-19 who are at high risk of clinical progression [169,170] (24 May 2021)	emergency use authorization [174] (21 November 2020)	can be used in patients not requiring oxygen and at high risk of progressing to severe disease [175] (26 February 2021)
Sotrovimab VIR-7831	No data found	emergency use authorization [135,176] (26 May 2021)	can be used in patients not requiring oxygen and at high risk of progressing to severe disease [177] (21 May 2021)
Tocilizumab	in combination; for hospitalized patients on high-flow oxygen or noninvasive ventilation who have evidence of clinical progression or increased markers of inflammation [164] (27 May 2021)	emergency use authorization [147] (24 June 2021)	No data found

## Data Availability

Data sharing not applicable.
